# Supplementary material for: OsTBP2.1, a TATA-Binding Protein, Alters the Ratio of OsNRT2.3b to OsNRT2.3a and Improves Rice Grain Yield
Source: Int J Mol Sci. 2022 Sep 16;23(18):10795. doi: 10.3390/ijms231810795 (PMC9503026; doi:10.3390/ijms231810795)
Supplement: Supplementary file 1 [file ijms-23-10795-s001.zip › ijms-1867587-supplementary.pdf]

Figure S1

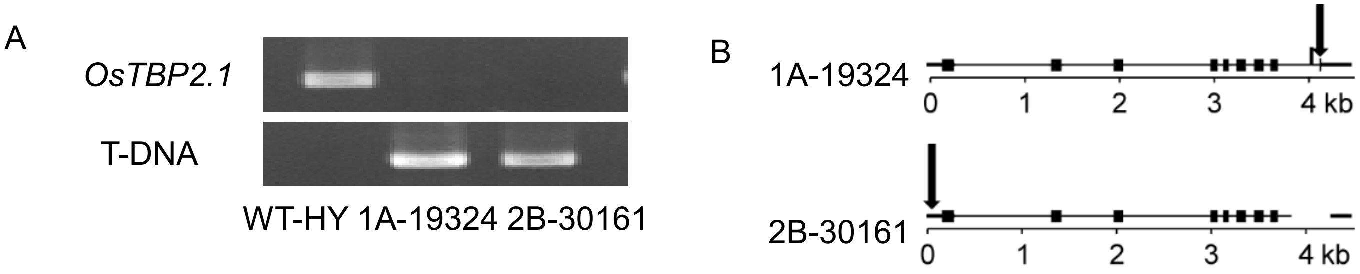
A
OsTBP2.1
T-DNA
WT-HY 1A-19324 2B-30161
B
1A-19324
0
1
2
3
4 kb
2B-30161
0
1
2
3
4 kb

Figure S2

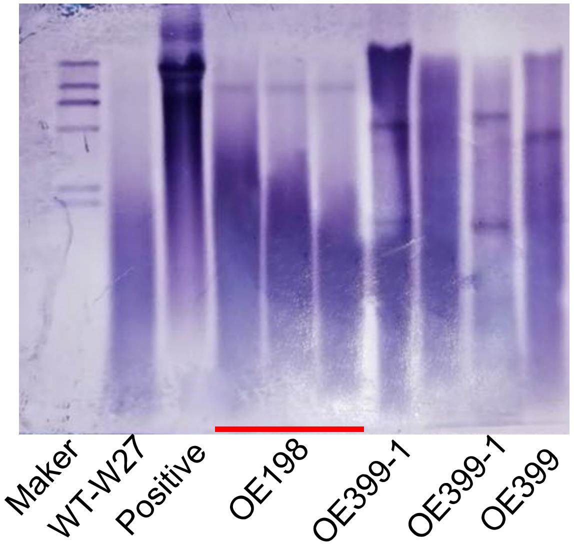

Figure S3

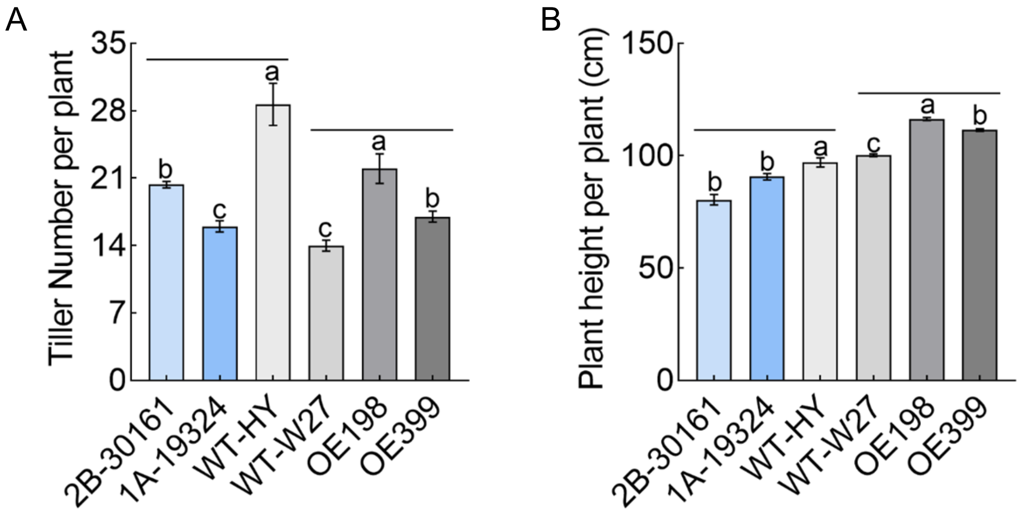

A
35
28
21
14
7
0
Tiller Number per plant
b
c
a
c
a
b
2B-30161
1A-19324
WT-HY
WT-W27
OE198
OE399
B
150
100
50
0
Plant height per plant (cm)
b
b
a
c
a
b
2B-30161
1A-19324
WT-HY
WT-W27
OE198
OE399

Figure S4

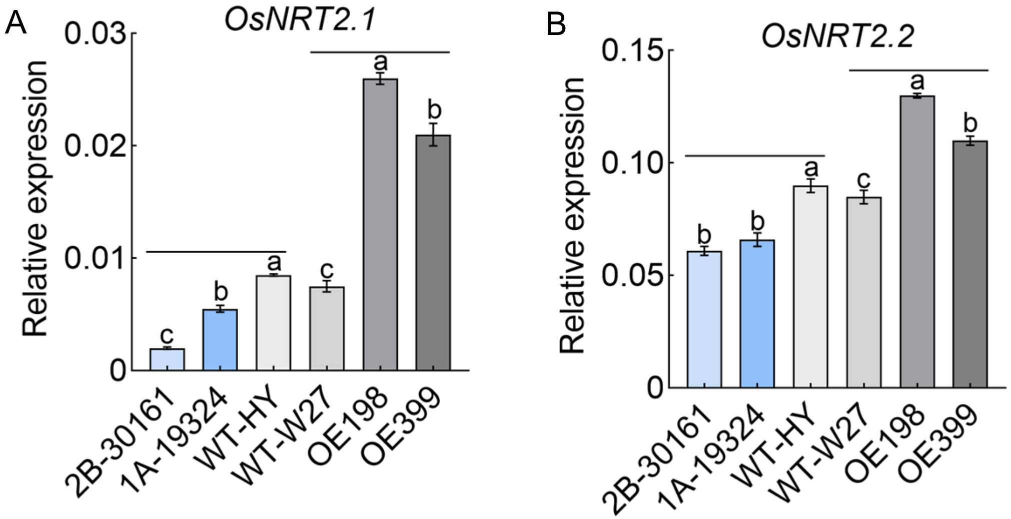
A
OsNRT2.1
Relative expression
0.03
0.02
0.01
0
a
b
c
a
b
c
2B-30161
1A-19324
WT-HY
WT-W27
OE198
OE399
B
OsNRT2.2
Relative expression
0.15
0.10
0.05
0
b
b
a
c
a
b
2B-30161
1A-19324
WT-HY
WT-W27
OE198
OE399

Figure S5

```
OsNRT2.3a  TCCACGTTCGCCGCGCCGCCGCTGCTGCCGCTCATCCGCGACACCCTCGGGCTCACGGCC
           |||||||||
OsNRT2.3b  TCCACGTTC...................................................
OsNRT2.3a  ACGGACATCGGCAACGCCGGGATCGCGTCCGTGTCGGGCGCCGTGTTCGCGCGTCTGGCC
                                                   |||||||||||||||||||||
OsNRT2.3b  .......................................GCCGTGTTCGCGCGTCTGGCC
OsNRT2.3a  ATGGGCACGGCGTGCGACCTGGTCGGGCCCAGGCTGGCCTCCGCGTCTCTGATCCTCCTC
           ||||||||||||||||||||||||||||||||||||||||||||||||||||||||||||
OsNRT2.3b  ATGGGCACGGCGTGCGACCTGGTCGGGCCCAGGCTGGCCTCCGCGTCTCTGATCCTCCTC
OsNRT2.3a  ACCACACCGGCGGTGTACTGCTCCTCCATCATCCAGTCCCCGTCGGGGTACCTCCTCGTG
           ||||||||||||||||||||||||||||||||||||||||||||||||||||||||||||
OsNRT2.3b  ACCACACCGGCGGTGTACTGCTCCTCCATCATCCAGTCCCCGTCGGGGTACCTCCTCGTG
     ZIIIB  CGCTTCTTCACGGGCATCTCCCTGCAGGTCGCCACATTAGCAATGCCACATTAGCAATGC
           ||||||||||||||||||||||||||||||||||||||||||||||||||||||||||||
     ZIIIB  CGCTTCTTCACGGGCATCTCCCTGCAGGTCGCCACATTAGCAATGCCACATTAGCAATGC
     ZIIIB  CGACTCTAGAGGATCCC
           |||||||||||||||||
     ZIIIB  CGACTCTAGAGGATCCC
```

Table S1 The primer sequences used in build vectors

	Primer name	Sequences (5' to 3')
Different length *OsNRT2.3* promoter lines	141 bp-F	aaAAGCTTACCCAACCATCGCGCCACGC
	180 bp-F	aaAAGCTTCTTTATTCATTTTCTCTCCAC
	243 bp-F	aaAAGCTTCGACGCGAGCGCGGAGACGG
	697 bp-F	aaAAGCTTGAGTAGATGAGAGAGAGGATG
	1505 bp-F	aaAAGCTTATATACCGTGAGATTTGATGC
	Different length-R	aaTCTAGACTCCAACACGTGGTAGCAAG
	OsNRT2.3-F	aaTCTAGAATGGAGGCTA AGCCGGTGGC
	OsNRT2.3-R	aaGGATCCATGCCCGTGA AGAAGCGCACG
pUbi::OsTBP2.1	*OsTBP2.1*-F	AAAGGATCCGGTTTTTGGGGGTGTGTAT
	OsTBP2.1-R	AAAGGTACCTTGCTGGACTTTTCTGAAC
OsTBP2.1 mutant lines	1A-19324-F	TGAAGACCGGACAATGGATC
	1A-19324-R	ATTATTGCCGGCAATAAGCC
	2B-30161-F	CTGCCTTACGAGCTGACCTC
	2B-30161-R	CATACACACCCCCAAAAACC

Table S2 The primer sequences used in real-time PCR

	Primer name	Sequences (5' to 3')
Different length *OsNRT2.3* promoter lines	*OsNRT2.3a*-F	GCTCATCCGCGACACCCT
	OsNRT2.3b-F	CACGTTCGCCGTGTTC
	OsNRT2.3a/b-ZIIIB-R	ATTGCTAATGTGGCGACCT
OsTBP2.1 transgenic lines	*OsNRT2.3*-F	CGCTGCTGCCGCTCATCCG
	OsNRT2.3-R	CCGTGCCCATGGCCAGAC
	OsTBP2.1-F	AATCAGGCTGGAGGGACTTG
	OsTBP2.1-R	TCAAAGGCGGTATACGTCTCA
	OsACTIN-F	CAACACCCCTGCTATGTACG
	OsACTIN-R	CATCACCAGAGTCCAACACAA
